# Polymorphisms of the SCN1A gene in children and adolescents with primary headache and idiopathic or cryptogenic epilepsy: is there a linkage?

**DOI:** 10.1007/s10194-011-0359-8

**Published:** 2011-06-29

**Authors:** Irene Toldo, Alice Bruson, Alberto Casarin, Leonardo Salviati, Clementina Boniver, Stefano Sartori, Pasquale Montagna, Pier Antonio Battistella, Maurizio Clementi

**Affiliations:** 1 Child Neurology Unit, Department of Pediatrics, University of Padua, Via Giustiniani, 3, 35128 Padua, Italy; 2 Genetic Unit, Department of Pediatrics, University of Padua, Padua, Italy; 3 Department of Neurological Sciences, University of Bologna, Bologna, Italy

**Keywords:** Migraine, Epilepsy, Comorbidity, *SCN1A* gene, Children

## Abstract

The purpose of this study was to evaluate the distribution of the polymorphisms of the SCN1A gene in a series of children and adolescents with primary headache and idiopathic or cryptogenic epilepsy compared to controls. Five non-synonymous exonic polymorphisms (1748A > T, 2656T > C, 3199A > G, 5771G > A, 5864T > C) of the SCN1A gene were selected and their genotyping was performed, by high resolution melting (HRM), in 49 cases and 100 controls. We found that among the five polymorphisms, only 3199A > G was a true polymorphism. We did not find a statistically significant difference between distribution of 3199A > G genotypes between cases and controls. We excluded the role of the SCN1A gene in the pathogenesis of comorbidity between headache (especially migraine) and epilepsy. The SCN1A gene is a major gene in different epilepsies and epilepsy syndromes; the HRM could be the new methodology, more rapid and efficacious, for molecular analysis of the SCN1A gene.

## Introduction

Migraine and epilepsy are both common neurological disorders and can occur as comorbid conditions [1–7]. From a genetic point of view, migraine is a polygenic multifactorial disorder [8], while many epilepsy syndromes are monogenic and have been pathogenetically linked to channelopathies [9]. Similarly familial hemiplegic migraine (FHM), the only known autosomal subtype of migraine, is due to mutations in genes encoding ion channels, and it is often associated to epileptic seizures [10]. Previous studies have excluded the role of the two FHM genes (*CACN1A*, *ATP1A2*) in the common forms of migraine [8, 11–13].

Considering the multifactorial etiology of migraine, genetic studies on phenotypically homogenous populations are required [8]. The comorbidity between two conditions that share common pathogenetic mechanisms, such as migraine and epilepsy, could address the issue.

The *SCN1A* gene encodes the α-subunit of the neuronal voltage-gated sodium channel Na_v_1.1 (MIM# 182389). Mutations in *SCN1A* are associated with a spectrum of epilepsy syndromes [14]; a few mutations result in FHM [15]. So far, *SCN1A* gene has never been investigated in subjects with common forms of migraine.

The purpose of this study was to evaluate the role of the *SCN1A* gene in the pathogenesis of comorbidity between primary headache and idiopathic or cryptogenic epilepsy in a large series of children and adolescents.

## Materials and methods

This was a case–control study comparing the genotype distribution and allele frequency of single nucleotide polymorphisms (SNPs) of *SCN1A* gene between patients and healthy controls.

Fifty-six subjects out of 1,795 headache sufferers under the age of 18 consecutively diagnosed at the juvenile Headache Center of the Department of Pediatrics of Padua Hospital between 1995 and 2008 were selected according to the following criteria: (1) primary headache and (2) idiopathic or cryptogenic epilepsy or unprovoked seizures. Patients with secondary headache or symptomatic epilepsy were excluded from the study. The methodology of patients’ recruitment and phenotype selection has been previously reported [7]. The study protocol and consent/assent forms were approved by our Institutional Review Board.

Forty-nine patients and 100 healthy controls participated in the genetic study. All the healthy controls were interviewed by a board certified child neurologist regarding, in particular, the personal history of headache; those affected by migraine or probable migraine, according to the criteria of the International Classification of Headache Disorders (ICHD-II) [16] were excluded.

The molecular analysis was conducted at the Genetic Laboratory of the Department of Pediatrics (University of Padua). Parental informed consent for genetic testing was obtained for each subject included in the study. Genomic DNA was extracted from peripheral blood using the High Pure PCR Template Preparation Kit (Roche Diagnostics GmbH, Mannheim, Germany).

The five SNPs examined are described in Table 1. Primers were designed using the Primer 3 software (http://frodo.wi.mit.edu/primer3). Primer sequences and amplification conditions for *SCN1A* gene are available upon request. Genotypes were determined by high resolution melt (HRM) analysis [17–19].

**Table 1:** Description of the polymorphisms of the SCN1A gene in this study

Base pair change	Aminoacid	Position	Frequency of heterozygous reported in literature	Clinical association	Reference
c.1748A > T	p.D583 V	Exon 11	ND	NK	NCBI
c.2656T > C	p.S886P	Exon 15	ND	NK	NCBI
c.3199A > G	p.T1067A	Exon 16	0.33	The SNP is more frequent in patients with epilepsy than in controls (33)	[24, 25, 27–33]
c.5771G > A	p.R1924H	Exon 26	ND	NK	NCBI
c.5864T > C	p.I1955T	Exon 26	0.025	NK	[41]

*ND* not determined, *NK* not known

The polymerase chain reaction (PCR) cycling and HRM analysis were carried out sequentially on a Rotor Gene 6000 (Corbett Research, Mortlake, Australia) using Takara Ex Taq R-PCR custom (Takara Bio Europe SAS, Saint-Germain-en-Laye France). The HRM analysis was performed with the temperature ramping (70 to 95°C, rising by 0.1°C) and fluorescence acquisition setting recommended by the manufacturer. The melting curves were normalized through calculation of the “line of best fit” of two normalization regions before and after the major fluorescence drop, corresponding to the melting of the PCR product using the software provided with the Rotor Gene 6000 (Corbett Research). Melting profiles and HRM running conditions were determined experimentally in 10 reference controls that were sequenced.

We obtained three different melting profiles only for the 3199A > G polymorphism. We sequenced two samples for each melting profile of each polymorphism, confirming that different profiles corresponded to different sequences. Sequence analysis revealed that heterozygotes and homozygotes have characteristic melting profiles that give rise to differently shaped melting curves. Therefore, in the following HRM analyses we used the curves of homozygotes and heterozygotes that were confirmed by sequencing as the reference for the genotype analysis of the unknown samples.

Direct sequencing of PCR products with abnormal HRM profiles was performed with the use of the BigDye^®^ Terminator v1.1 Cycle Sequencing Kit (Applied Biosystems) on ABI 310 automated DNA sequencer (PE Applied Biosystems, Foster City, CA, USA). The HRM profiles of cases with comorbidity were compared to HRM profiles of healthy reference controls that were sequenced.

The allele frequencies and genotypes in patients and controls were calculated. Genotype distributions in patients and controls were tested for Hardy–Weinberg equilibrium, and differences in genotype frequencies between patients and controls were tested using a Chi-square test for independence, with a level of significance set at 0.05.

## Results

Among 1,795 headache sufferers under the age of 18 consecutively diagnosed at the juvenile Headache Center of the Department of Pediatrics of Padua Hospital between 1995 and 2008, fifty-six subjects having comorbidity between primary headache and idiopathic or cryptogenic epilepsy or unprovoked seizures were selected. The clinical characteristics and the statistical analysis of these two population study have been previously reported [7].

The present study focuses on the results of the *SCN1A* gene molecular analysis, which was undertaken in 49 cases and 100 healthy controls. Among the 49 cases, 45 subjects (92%) had migraine (M) (38 migraine without aura, 7 migraine with aura) and 4 patients suffered from episodic tension type (ETTH) headache. Considering the type of epilepsy, there were 30 subjects with focal cryptogenic epilepsy (27 M, 3 ETTH), 11 with focal idiopathic epilepsy (10 M, 1 ETTH), and 8 (8 M, 0 ETTH) with idiopathic generalized epilepsy.

**Fig. 1:**
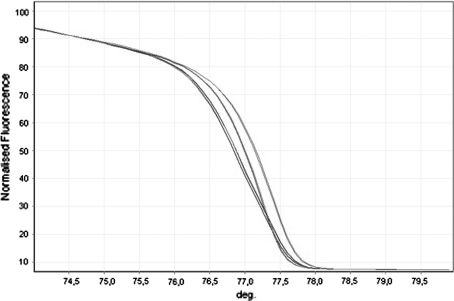
Rotor Gene 6000 HRM normalized graph of the region amplified for exon 16. SCN1A 3199A > G polymorphism; *green*, homozygous for the A allele (AA); *red*, homozygous for the G allele (GG); *blue*, heterozygous (AG)

**Fig. 2:**
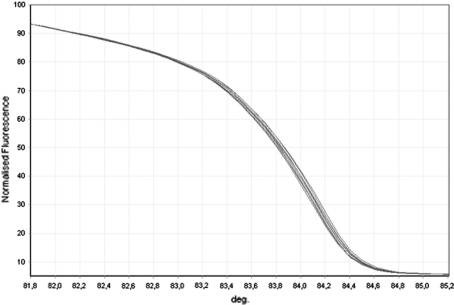
Rotor Gene 6000 HRM normalized graph of the region amplified for exon 11. SCN1A 1748A > T polymorphism; *red* (sequenced reference controls), homozygous for the A allele (AA); *green* (samples examined), homozygous for the A allele (AA)

The HRM profiles consist of three different graphs (melt temperature, normalized graph and the difference graph) that are visualized all together during the analysis of results. As an example, we report the three graphs concerning the HRM analysis of exon 16 (Fig. 1) and of exon 11 (Fig. 2).

Comparing the HRM profiles of the cases and healthy control samples with those of the sequenced reference controls, we could identify the genotypes of the samples examined.

The polymorphism 3199A > G, having a significant heterozygosity (0.33), exhibited three HRM profiles consistent with the three genotypes (AA, GA, GG), as confirmed by direct sequencing. The other four SNPs having a low or not known heterozygosity disclosed a unique HRM profile, consistent with the wild type genotype.

During HRM analysis of exon 11, we found in one patient a different profile. The PCR product was analyzed by direct sequencing that disclosed a missense variation sequence 1811G > A that causes at protein level a replacement of arginine with histidine (R604H). The patient is a 15-year-old boy with a history of childhood absence epilepsy (since 3–6) suffering from episodic tension headache, since the age of 11. He never experienced febrile seizures or periodic syndromes. The analysis was carried out in both the parents; the mother was negative while the father, who was asymptomatic, carried the missense variation sequence 1811G > A.

During the analysis of healthy controls, we found in one case a missense variation sequence 5782C > G that causes at protein level a replacement of arginine with glycine (R1928G).

The allele and genotype frequencies, respectively, absolute and percentage, of the five SNPs observed in 49 patients and 100 healthy nonmigrainous controls, tested by HRM, are presented in Table 2.

**Table 2:** Allele and genotype frequencies, respectively absolute and percentage (in brackets), of the five SNPs of the *SCN1A* gene observed in 49 cases and 100 controls, tested by HRM

	SNPs of the SCN1A gene				
	1748A > T	2656T > C	3199A > G	5771G > A	5864T > C
Cases (*N* = 49)					
Allele frequencies	A 98 (100%)	T 98 (100%)	A 65 (66.3%)	G 98 (100%)	T 98 (100%)
	T 0 (0%)	C 0 (0%)	G 33 (33.7%)	A 0 (0%)	C 0 (0%)
Genotype frequencies	A/A 49 (100%)	T/T 49 (100%)	A/A 22 (44%)	G/G 49 (100%)	T/T 49 (100%)
	A/T 0 (0%)	T/C 0 (0%)	A/G 21 (44.7%)	G/A 0 (0%)	T/C 0 (0%)
	T/T	C/C	G/G	A/A	C/C
	0 (0%)	0 (0%)	6 (11.3%)	0 (0%)	0 (0%)
Controls (*N* = 100)					
Allele frequencies	A 200 (100%)	T 200 (100%)	A 123 (61.5%)	G 200 (100%)	T 200 (100%)
	T 0 (0%)	C 0 (0%)	G 77 (38.5%)	A 0 (0%)	C 0 (0%)
Genotype frequencies	A/A 100 (100%)	T/T 100 (100%)	A/A 42 (37.8%)	G/G 100 (100%)	T/T 100 (100%)
	A/T 0 (0%)	T/C 0 (0%)	A/G 39 (47.4%)	G/A 0 (0%)	T/C 0 (0%)
	T/T 0 (0%)	C/C 0 (0%)	G/G 19 (14.8%)	A/A 0 (0%)	C/C 0 (0%)

The analysis of the frequency distribution showed that, only for the polymorphism 3199A > G, the two alleles (A: wild type; G: mutated) are represented both in cases and controls; while for the other four SNPs the allele frequency of the wild type was 100% both in cases and controls, as assessed on a total of 298 alleles, therefore these SNPs are not true polymorphisms in the tested population. In the case of polymorphism 3199A > G, the genotype distributions for the two alleles (A and G) were in equilibrium under the Hardy–Weinberg law both in cases (*p* = 0.64) and controls (*p* = 0.44) (with Chi-square test the balance is present if *p* > 0.05). For this polymorphism, the comparison of the distribution of allele (A, G) and genotype (A/A, A/G, G/G) frequencies in cases and controls by Chi-square test was not statistically significant (respectively, *p* = 0.49, *p* = 0.58).

## Discussion

Previous studies have shown that cases affected by FHM, carrying mutations of genes encoding ion channels (*CACNA1A*, *ATP1A2*, *SCN1A*), may have seizures [20–22].

Molecular analysis of the *CACNA1A* and *ATP1A2* genes in patients with migraine without aura and with aura, has shown that these two genes are not involved in common forms of migraine [8, 11–13], although similar studies have not examined the *SCN1A* gene.

The comorbidity between migraine and epilepsy may allow to select a clinically more homogeneous subgroup of patients in order to carry out genetic studies more targeted than those conducted in patients with common forms of migraine. This is confirmed by the study of Deprez et al*.* [23], carried out on 20 families with migraine and epilepsy, which showed mutations in the *ATP1A2* gene in 10% of cases, suggesting to perform molecular analysis of this gene in cases where there is comorbidity between migraine and epilepsy and positive family history for migraine or epilepsy.

In our study, molecular analysis of five polymorphisms in coding exons of the *SCN1A* gene was performed on a large series consisting of 49 subjects with comorbidity between primary headache and idiopathic or cryptogenic epilepsy and 100 healthy nonmigrainous controls.

Among the five SNPs analyzed, only 3199A > G was confirmed to be a polymorphism while the other four SNPs were not true polymorphisms, because they were not found in the 298 alleles (cases and controls) examined and they are not to be further investigated.

We did not find a statistical significant difference of 3199 A > G genotypes’ distribution between cases and controls; therefore our results confirm that the polymorphism 3199A > G is not associated to pathological phenotypes in headache sufferers and in migraineurs, similar to that found in other studies conducted on patients with epilepsy.

From a statistical point of view, the group sample sizes achieve a power lower than 20% to detect, as statistically significant, the observed differences between the two groups for both allele and genotype analyses for the polymorphism 3199A > G. Actually, those differences could be considered negligible from a genetic point of view when bearing in mind the epidemiological and clinical characteristics of the disease. Furthermore, specifically in reference to genotypes, the observed prevalence would suggest that the A/A genotype is less prevalent among wild type subjects, and this would be inconsistent with expected. For these reasons, in our opinion, the results suggest that the observed differences are due to chance.

To our knowledge, this is the first study that has assessed the genotype of polymorphisms in the *SCN1A* gene in cases with comorbidity between headache and epilepsy.

The *SCN1A* gene has so far been well studied in large series of patients with severe myoclonic epilepsy [24–26] or in families with idiopathic generalized epilepsies and febrile seizures plus (GEFS+) [27, 28] and also in some families with febrile seizures [29].

These studies also contain data on the frequency of polymorphism 3199A > G of *SCN1A* gene, and documented genotypic and allelic frequencies comparable between patients and controls. The polymorphism 3199A > G was analyzed specifically in patients with febrile convulsions [30] or with migrainous vertigo [31] excluding a positive correlation.

In two recent studies, the analysis of polymorphism 3199A > G of the *SCN1A* gene was conducted in Asiatic patients affected by epilepsy [32, 33]; in both studies genotype distributions in drug-responsive and drug-resistant patients did not differ significantly.

Only in the study of Lakhan et al*.* [33] a statistically significant correlation between the AG genotype of polymorphism 3199G > A and the presence of epilepsy in cases compared to controls has been found; however, this finding deserves further study because the statistical significance was observed only in heterozygous subjects, and thus it is more difficult to explain in terms of biological plausibility. Overall, the results of the studies conducted so far argue that the polymorphism 3199A > G of the *SCN1A* gene is not associated with pathological phenotypes; our results confirm that this also applies to headache and migraine sufferers.

We found a missense mutation 1811G > A (R604H) in exon 11 in a patient with episodic tension headache and childhood epilepsy with absences; in the literature this mutation is already known [34–36]. Escayg et al. [34] identified the same mutation in two patients with juvenile myoclonic epilepsy and emphasized that this mutation interrupts a consensus site for protein kinase A, which is conserved in the four major sodium channel of the central nervous system. However, this mutation did not cosegregate with the epileptic phenotype in two families with juvenile myoclonic epilepsy [34] and had a minor effect on ionic currents in the study by Smith et al*.* [37]. The possibility that this mutation is not pathogenic has been supported by two subsequent works [35, 36]. In our case, the pathogenetic significance of this mutation was excluded by carrying out the genetic analysis of the patient’s parents; in fact the father was asymptomatic and carried the same sequence variation.

We also confirm that the missense variant 5782C > G (R1928G), found in a single healthy nonmigrainous control among the 100 examined, is a rare missense polymorphism, as previously reported [27, 36].

Our study seems to exclude a role of the *SCN1A* gene in the pathogenesis of migraine and in particular in cases with comorbidity between migraine and epilepsy. The major limitation of the present study is that the genetic analysis has been focused on a part of the gene, since a complete genetic analysis seemed too expensive, given the large number of exons (equal to 26) of the *SCN1A* gene and based on the negative results in previous studies [27, 38].

Our data support the hypothesis [39], that the *SCN1A* gene is not implicated in the pathogenesis of common forms of migraine, even when the phenotype is restricted by the presence of comorbidity with epilepsy.

The *SCN1A* gene has a causative role in some epilepsy syndromes (severe myoclonic epilepsy and GEFS+) and has recently been associated with new and different clinical phenotypes, such as focal and generalized cryptogenic epilepsy [35] and atypical Panayiotopoulos syndrome [40].

Another important aspect of this study was the use of an innovative method, the HRM, for the molecular analysis of the *SCN1A* gene. The HRM offers significant advantages over traditional methods (SSCP, DGGE, DHPLC, etc.), as it does not require manipulation of genetic material after amplification, it is inexpensive, it has excellent sensitivity and specificity and allows the concomitant analysis of a large number of samples, increasing the reproducibility of analysis and significantly reducing the work time.

The sensitivity and specificity of variation detection of HRM are estimated, respectively, to be 100 and 95% [19].

These characteristics are of particular relevance, especially in the genetic analysis of multifactorial diseases, such as headache and epilepsy, which requires a large number of subjects and very often involves the analysis of several polymorphisms in the same patients. The HRM could then form the new method of molecular analysis of the *SCN1A* gene, which is currently usually performed with screening techniques (DHPLC) and direct sequencing.

## Conclusions

To the best of our knowledge, this is the first study that evaluated the association between polymorphisms of the *SCN1A* gene and comorbidity between primary headache (especially migraine) and epilepsy.

Our results confirm that the polymorphism 3199A > G is not associated with pathological phenotypes in patients with headaches and migraine, while the other four polymorphisms examined were homozygous in the tested populations and should not be further investigated.

We exclude the role of the *SCN1A* gene in the pathogenesis of comorbidity between headache (especially migraine) and epilepsy.

The *SCN1A* gene is a major gene in different epilepsies and epilepsy syndromes, and in this field it has to be further investigated. The HRM could be a new methodology, more rapid and efficacious, for molecular analysis of the *SCN1A* gene.

The development of protocols for more efficient and reliable molecular diagnostics would allow a wider appreciation of the role of the *SCN1A* gene and of other genes encoding for ion channels in different epilepsies and epilepsy syndromes.
